# Louisville Medical Institute

**Published:** 1841-12

**Authors:** 


### LOUISVILLE MEDICAL INSTITUTE.

In our last number we spoke of the prospects of a large class in the Medical Institute of Louisville as being encouraging. We are now able to state, for the satisfaction of its friends at a distance, that the expectation has been fully realized. The increase upon the number of students that were in attendance on the first of December, 1840, is more than twenty-five per cent. It will be found that the growth, this season, is fully equal to that of any former year, and that the class is one of the largest that ever assembled in any medical school west of the mountains. Y.

